# Atypical Rearrangements in APL-Like Acute Myeloid Leukemias: Molecular Characterization and Prognosis

**DOI:** 10.3389/fonc.2022.871590

**Published:** 2022-04-12

**Authors:** Luca Guarnera, Tiziana Ottone, Emiliano Fabiani, Mariadomenica Divona, Arianna Savi, Serena Travaglini, Giulia Falconi, Paola Panetta, Maria Cristina Rapanotti, Maria Teresa Voso

**Affiliations:** ^1^ Department of Biomedicine and Prevention, University of Tor Vergata, Rome, Italy; ^2^ Santa Lucia Foundation, Istituto di Ricovero e Cura a Carattere Scientifico (I.R.C.C.S.) Neuro-Oncohematology, Rome, Italy; ^3^ Department of Biomedicine and Prevention, UniCamillus‐Saint Camillus International University of Health Sciences, Rome, Italy; ^4^ Department of Experimental Medicine, Tor Vergata University of Rome, Rome, Italy

**Keywords:** variant acute promyelocytic leukemia, APL-like acute myeloid leukemia, atypical rearrangements, complex rearrangements, genetic landscape

## Abstract

Acute promyelocytic leukemia (APL) accounts for 10–15% of newly diagnosed acute myeloid leukemias (AML) and is typically caused by the fusion of promyelocytic leukemia with retinoic acid receptor α (*RARA*) gene. The prognosis is excellent, thanks to the all-trans retinoic acid (ATRA) and arsenic trioxide (ATO) combination therapy. A small percentage of APLs (around 2%) is caused by atypical transcripts, most of which involve *RARA* or other members of retinoic acid receptors (*RARB* or *RARG*). The diagnosis of these forms is difficult, and clinical management is still a challenge for the physician due to variable response rates to ATRA and ATO. Herein we review variant APL cases reported in literature, including genetic landscape, incidence of coagulopathy and differentiation syndrome, frequent causes of morbidity and mortality in these patients, sensitivity to ATRA, ATO, and chemotherapy, and outcome. We also focus on non-RAR rearrangements, complex rearrangements (involving more than two chromosomes), and NPM1-mutated AML, an entity that can, in some cases, morphologically mimic APL.

## Introduction

Acute promyelocytic leukemia (APL) was first described in 1957 by Hillestad and accounts for 10–15% of newly diagnosed acute myeloid leukemias (AML), with an incidence of about 1,900 cases per year in Europe (0.12 per 100,000 person-years) (1, 2). APL is characterized by the t(15;17)(q24.1;q.2) balanced translocation, which results in the fusion of promyelocytic leukemia (*PML*) with retinoic acid receptor α (*RARA*) gene. This oncogene, found in 98% of cases, causes the transcriptional repression of *RARA*-targeted genes and the destruction of PML nuclear bodies (PML-NBs), resulting in altered self-renewal, senescence mechanisms, and response to DNA damage (3–7). With the introduction of daunorubicin in 1973, all-trans-retinoic acid (ATRA) in 1988, arsenic trioxide (ATO) in 1997, and, eventually, the chemo-free ATRA+ATO approach in 2006, the disease changed from highly fatal to curable leukemia in most cases (6, 8–14).

Morphologically, APL is characterized by the presence, in the bone marrow (BM) and/or peripheral blood (PB), of immature hypergranular promyelocytes with abundant cytoplasm, irregular nuclei with fine azurophilic granules, and Auer rods in 90% of cases. The immunophenotypic evaluation often shows the expression of myeloid antigen CD13, CD33, CD117, and MPO, while CD34 and HLA-DR, as well as markers of granulocytic differentiation, results are absent or low. A rapid diagnosis of APL may be performed by analyzing the immunocytochemical pattern of the PML protein using the anti-PML monoclonal antibody PG-M3 (15, 16). However, a genetic confirmation of the *PML/RARA* fusion transcript is mandatory and is carried out by RT-PCR or RT-QLAMP, and it should be performed by turnaround times of 24–48 h in order to warrant a rapid treatment start, which is associated with a reduction in the rate of bleeding complications, the major cause of early death in APL (15, 17–19). Other diagnostic approaches, such as conventional karyotyping and FISH, are useful to identify the t(15;17) translocation (15).

About 2% of APL are characterized by atypical rearrangements, where the RARA is fused to partners other than PML or in which the translocation involves other members of the RAR superfamily (**Table 1**) **(** 6). These APL-like forms are a tough challenge for the clinician, both because of the difficulty of diagnosis and the generally unfavorable outcome due to diagnostic delays and to the frequent resistance of these forms to treatment commonly used for classical APL (3, 4, 101, 102).

**Table 1:** Genetic, clinical, and prognostic features of variant and acute promyelocytic leukemias.

Fusion genes	Cases (N)	Cytogenetic	Coagulopathy	ATRA	ATO	Chemotherapy	Combination therapy	Differentiation Syndrome	Outcome (OS)	References
**RARA rearrangements**										
PML-RARA	98% of total	t (15;17) (q22;q21)	65-83%	S	S	S	S	2–54%	12 y, OS 87%	(13, 20–25)
ZBTB16-RARA	1% of total	t (ll;17) (llq23;q21)	40-55%	R	R	S	S	Not reported	25 mo; 40% alive	(16, 26–28)
STAT5B-RARA	17	t (17;17) (q21;q21)	8 (47%), 7 ND	R	R	S	S	0	15 mo (0–53); 7 alive; 7 dead; 3 ND	(29–42)
NPM1-RARA	11	t (5;17) (5q35;q21)	2 (18%), 8 ND	S	ND	S	S	0, 3 ND	18 mo OS (0–46); 7 alive; 2 dead; 2 CR, ND on FU	(28, 43–52)
IRF2BP2-RARA	6	t (l;17) (q42;q21)	2 (33.3%)	S	ND	ND	S	0	17 mo (1–50); 3 alive; 3 dead	(53–58)
TBLR1-RARA	4	t (3;17) (q26;q21)	0, 2 ND	ND	ND	ND	S	0	5.5 mo (0–11); 2 dead; 1 CR no data on FU; 1 ND	(59, 60)
FIP1L1-RARA	4	t (4;17) (ql2;q21)	0, 2 ND	S	ND	R	ND	2 (50%), 1 ND	2 mo OS (0–5); 1 alive; 2 dead; 1 CR ND on FU	(61–64)
BCOR-RARA	2	t (X;17) (pll;q21)	1 (50%)	ND	R	S	S	0	26.5 mo (26–41); 2 alive	(65, 66)
STAT3-RARA	2	t (17;17) (q21;q21)	ND	R	R	ND	R	0	20 mo (7–33); 2 dead	(67)
PRKAR1A-RARA	2	t (17;17) (q21;q24)	1 (50%)	ND	ND	ND	S	0	24 mo, alive; CR, ND on FU	(68, 69)
OBFC2A-RARA	1	t (2;17) (q32;q21)	0	ND	ND	S	S	0	15 mo, alive	(70)
6TF2I-RARA	1	t (7;17) (qll;q21)	1 (100%)	R	R	R	R	0	5 mo, dead	(71)
FNDC3B-RARA	1	t (3;17) (q26;q21)	1 (100%)	ND	ND	S	ND	1 (100%)	CR, ND on FU	(72)
NUP98-RARA	1	ND	1 (100%)	ND	ND	S	ND	0	44 mo, alive	(73)
TNRC18-RARA	1	ND	1 (100%)	R	R	S	ND	0	9 mo, alive	(74)
HNRNPC-RARA	1	t (14;17) (qll;q21)	0	R	ND	S	ND	0	12 mo, alive	(75)
X-RARA	1	t (X;17) (q28;ql2)	ND	ND	ND	S	R	0	23 mo, dead	(76)
**RARB rearrangements**										
TBLR1-RARB	6	t (3;3) (q24;q26)/inv.(3)/t (3;10;12)	1 (17%), 5 ND	R	ND	S	ND	ND	63 mo (23–108); 4 alive; 2 ND	(77–79)
X-RARB	1	t (X;3) (q28;q21)	ND	R	ND	S	ND	ND	31 mo, alive	(79)
**RARG rearrangements**										
CPSF6-RAR6	8	t (12;12) (ql3;ql5)	4 (50%), 3 ND	R	R	S	ND	1 (12.5%)	11 mo (1–33); 3 alive; 3 dead; 2 ND	(27, 77, 80–83)
NUP98-RAR6	5	t (ll;12) (pl5;ql3)	1 (20%), 1 ND	R	R	S	S	1 (20%)	20 mo (0-32); 1 alive; 3 dead; 1 CR ND on FU	(84–89)
PML-RAR6	1	t (12;15) (ql3;q22)	0	R	ND	S	ND	0	CR, ND on FU	(89)
NPM1-RAR6-NPM1	1	ND	0	R	R	ND	ND	0	8 mo, dead	(90)
HNRNPC-RAR6	1	ND	0	R	R	S	ND	0	13 mo, dead	(91)
**Non-RAR rearrangements**										
ELL-MLL/MLL-ELL	2	t (ll;19) (q23;pl3.3)	1 (50%), 1 ND	ND	ND	ND	S	ND	170 mo, alive; 1 ND	(77, 92)
MLL-AF1Q	1	t (l;ll) (q21;q23)	ND	ND	ND	ND	S	ND	34 mo, alive	(77)
RPRD2-MLL	1	t (l;ll) (q21;q23)	ND	ND	ND	ND	S	ND	34 mo, alive	(77)
NPM1-CCDC28A	1	ND	ND	ND	ND	ND	S	ND	54 mo, alive	(77)
TBC1D15-RAB21	1	ND	ND	ND	ND	ND	S	ND	56 mo, alive	(77)
**Complex rearrangements**										
TF6-RARA	1	14;17) (ql2;qll;q21)	0	S	ND	ND	S	0	3 mo, alive	(93)
PML-RARA	1	L7;15) (_P_13;q21;q22)	ND	S	ND	ND	ND	0	CR, ND on FU	(94)
PML-RARA	1	15;17) (q24;q24;qll)	ND	ND	ND	ND	S	0	4 mo, alive	(95)
PML-RARA	1	t (l;17;15) (q21;q21; q24)	1 (100%)	ND	ND	ND	S	1 (100%)	48 mo, alive	(96)
PML-RARA	1	t (6;17;lS) (p21;q21; q22)	1 (100%)	S	S	ND	ND	0	60 mo, alive	(97)
PML-RARA	1	17;15) (q25;q21;q24)	1 (100%)	ND	ND	S	ND	0	4 mo, alive	(98)
PML-RARA	1	t (5;17;15;20) (q33;q12;q22;qll.2)	1 (100%)	ND	ND	ND	S	0	CR, ND on FU	(99)
PML-RARA	1	34;q21;q24;q13;q 26.1)	1 (100%)	ND	ND	ND	S	1 (100%)	CR, ND on FU	(100)

CR, complete response; FU, follow-up; mo, months; ND, no data; OS, overall survival; R, resistant; S, sensitive.

This paper focus on the latter entities in order to summarize knowledge on the molecular landscape and correlations with outcome.

Variant APLs usually have a clinical presentation and morphological and immunophenotypic picture similar to classical APL, including pancytopenia-related symptoms (weakness, fatigue, infection) and bleeding (103). Therefore, if genetic tests result negative or are not readily available, a FISH analysis using RARA, RARB, and RARG probes or conventional karyotype helps to identify possible alternative RAR translocations or other genetic abnormalities. In this context, the morphologic and/or immunophenotypic features of some NPM1-mutated AMLs also resemble APL and will be described in this review.

## Pathogenesis, Characteristics, and Management of APL Variants

The pathogenesis of APL has been extensively studied and discussed in previous papers. The chromosomal translocation t(15;17), resulting in the PML-RARA fusion protein, is the main and, probably, the only driver alteration of APL, where additional gene mutations have been reported at a significantly lower rate when compared to other AML subtypes (26, 104, 105). The differentiation block typical of APL results from the destruction of PML nuclear bodies, implicated in DNA replication, transcription, and epigenetic silencing, and from the repression of RARA target differentiation genes by the aberrant recruitment of histone deacetylases (2–4, 105, 106).

**Figure 1:**
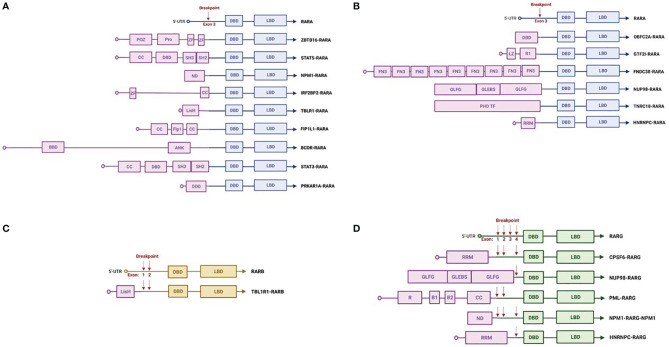
Schematic representations of *RARx* translocations. Common **(A)** and rare **(B)** *RARA* rearrangements. *RARB* **(C)** and *RARG* **(D)** rearrangements. The figures were created with Biorender.com. 5′-UTR, 5′-untranslated region; DBD, DNA-binding domain; LBD, ligand-binding domain; CC, coiled coil domain; POZ, BTB/POZ domain; Pro, proline-rich region; Zn, zinc finger domain; SH3, protein–protein interaction domain; SH2, docking domain for phosphorylated tyrosine residues; BBD, BCOR Bcl6-binding domain; ANK, ankyrin repeats; Fip1, FIP1-binding domain for polymerase; LisH, lissencephaly type-1-like homology motif; DDD, dimerization/docking domain of the type I alpha regulatory subunit of cAMP-dependent protein kinase; ND, nucleoplasmin/nucleophosmin domain; LZ, leucine zipper; PHD TF, plant homeodomain finger transcription factor domain; FN3, fibronectin type 3 domain; GLEBS, Gle2/Rae1-binding sequence; GLFG, Gly-Leu-Phe-Gly repeats; R1, I-repeat domains; RRM, RNA recognition motif; R, RING finger domain; B1 and 2, B box.

Several rearrangements different from PML-RARA have been described as driver mutations in APL-like AML (**Figure 1**). In most cases, the RAR gene family is involved (1, 103), including RARB and RARG in addition to RARA, all with a key role in cell development and differentiation (2, 107). Few cases of rearrangements other than RAR, mainly pediatric, have been reported (3, 77).

We reviewed the most frequently reported genetic alterations in APL-like AML since 2010, including cases identified with modern diagnostic standards and treated with current therapeutic strategies (**Table 1**). Because of clinical relevance, we report data on coagulopathy (main cause of death in APL patients) and differentiation syndrome (DS) (13, 20–22). Coagulopathy is defined as prolonged prothrombin time and/or activated partial thromboplastin time in addition to hypofibrinogenemia and/or increased levels of fibrin degradation products or D-dimer (23–25). We excluded from our analysis cases in which a rearrangement was not identified or cases with a cryptic PML-RARA rearrangement.

### RARA Rearrangements

In APL variants, rearrangements involving *RARA* gene are the most frequently described. The most common translocation, reported in 1% of cases, is the t(11;17)(q23;q21), which fuses *RARA* with *ZBTB16* (formerly known as promyelocytic leukemia zinc finger protein PLZF), a key regulator of physiological and stress-induced myelopoiesis (1–3, 108). Of note is that both the oncogenic fusion transcripts *PML-RARA* and *ZBTB16-RARA* are characterized by the identical *RARA* exon sequence, suggesting a key role of RARA domains in the pathogenesis of this disease. In contrast to classic APL (109, 110), characterized by a low number of additional mutations, this variant presents a complex molecular landscape, similar to other acute myeloid leukemias (AML) (109, 110). Our group showed that *ZBTB16-RARA* rearranged AML and display several gene mutations commonly identified in AML, including *TET2*, *RUNX1*, and *CSF3R*, and in particular, the most frequent alteration was found in the AT-rich interacting domain containing protein 1A gene (*AIRD1A*, in 6 out of 7 patients analyzed), a member of the SWI/SNF family of transcriptional regulators, which is a known solid and hematological cancer development driver (26, 111).

Data in literature report a lower incidence of coagulopathy compared to classic APL (40–55%) and a poor outcome, with less than half of the patients surviving at prolonged follow-up (26–28, 102). The therapy with ATRA and ATO is not effective on ZBTB16-RARA AML, and the resistance to ATO is due to the lack of an ATO-binding site, whereas although ATRA induces the degradation of the fusion protein, differentiation and apoptosis do not occur, and there is no clinical response. Indeed DS has been reported in none of the cases (8, 9, 112, 113). The proteolytic activity of ATRA has shown positive results in combination with chemotherapy, which remains the main therapeutic strategy in these patients.

The recent finding of the high prevalence of ARID1A mutations might open new scenarios on the use of targeted drugs since this mutation has been associated with a decrease in intracellular glutathione (GSH). Eprenetapopt (APR-246), a GSH p53-targeting compound inhibitor, has been shown to be effective in both ARID1A-mutated solid tumors and p53-mutated myelodysplastic syndromes and may be active in ZBTB16-RARA AML (10, 11, 114–116).

The signal transducer and activator of transcription 5B (STAT5B) is a member of the STAT family transcription factors and mediates cell differentiation, proliferation, and survival signals induced by various cytokines and hormones (13, 117). STAT5 has been linked to several hematological cancers, in particular lymphoma and acute leukemia (118). Fusion with RARA generates the t(17;17)(q21;q21) and the *STAT5B-RARA* rearrangement, of which 17 cases have been described (29–41). This variant shares common features with ZBTB16-RARA AML: a moderate incidence of coagulopathy, resistance to ATRA and ATO, and poor outcome (42). Chemotherapy is likewise the best strategy, but hypomethylating agents may also be effective, as shown in a case successfully treated with decitabine and ATRA (29). Given the high relapse rate, patients with STAT5B/RARA-positive leukemia might benefit from hematopoietic stem cell transplantation in the first remission.

The rearrangement of *RARA* with nucleophosmin 1 [*NPM1-RARA*, t(5;17)(5q35;q21)], a protein physiologically implicated in genomic stability and DNA repair and one of the most commonly mutated genes in AML, has been frequently reported (101, 102, 119, 120).

These cases have morphologic and phenotypic features similar to PML-RARA-positive APL, but blasts display abundant cytoplasm with small azurophilic granules and regular nuclear outline. A coagulopathy has been rarely reported (2 out of 11 cases), and the prognosis seems better than for the previous variants, probably because of the sensitivity to the drugs used in classic APL. Indeed NPM1-RARA-positive AML are sensitive to ATRA (alone or in combination with chemotherapy, but DS has not been reported (28, 43–52)).

Interferon regulatory factor 2-binding protein 2 (IRF2BP2) plays a role in apoptosis, survival, and cell differentiation (121). In six cases of t(1;17)(q42;q21), *IRF2BP2-RARA* **-**rearranged AML has been reported. Coagulopathy occurred in 2 patients; ATRA, alone and in combination with chemotherapy, resulted effective, while no DS has been reported (53–58).

Transducin β-like receptor 1 (TBLR1) plays an important role in stem cell proliferation and differentiation and is involved in the oncogenesis of solid tumors (122). The *TBLR1-RARA* fusion transcript [t(3;17)(q26;q21)] has been described in 4 cases. ATRA and ATO were successfully used in combination with chemotherapy, and no coagulopathy and DS were observed (59, 60).

Factor interacting with PAPOLA and CPSF1 (FIP1L1), fused with platelet-derived growth factor receptor α (PDGFRα), has been linked to several hematologic malignancies (hypereosinophilic syndrome, chronic eosinophilic leukemia, systemic mastocytosis) (123). The FIP1L1-RARA chimeric protein [t(4;17)(q12;q21)]-positive AML is ATRA-sensitive, and two DS events were reported. Nevertheless, it is an aggressive variant, and half of the reported cases died due to bleeding before the start of therapy or resistance first to chemotherapy and then to ATRA+chemotherapy. No coagulopathy has been reported (61–64).

BCL6 Corepressor (BCOR) is an epigenetic regulator whose alterations recur in solid and hematologic tumors (124). Two *BCOR-RARA* [t(X;17)(p11;q21)]-positive AML cases have been described. One patient presented coagulopathy that was relieved by ATRA and tamibarotene. Both patients underwent chemotherapy with and without ATRA, and complete response was achieved in both cases. The role of ATRA as monotherapy is therefore uncertain, and ATO resulted ineffective (65, 66).

Signal transducer and activator of transcription 3 (STAT3), a member of the STAT family transcription factors, has functions similar to STAT5 and has been likewise linked to hematological cancers (118). A *STAT3-RARA* variant [t(17;17)(q21;q21)] has been reported in two cases with unfavorable outcome. Both patients were resistant to ATRA, and one of them was resistant to ATO. One patient, treated with chemotherapy, achieved a temporary complete response (67).

The rearrangement between RARA and protein kinase CAMP-dependent type I regulatory subunit alpha PRKAR1A-RARA, [t(17;17)(q21;q24)], implicated in several cellular functions and in the tumorigenesis of solid cancers, has been described in two cases (125). Both were sensitive to ATRA in combination with chemotherapy (19). One patient presented coagulopathy at onset (68, 69).

Individual cases of rare rearrangements of RARA with the following genes have been described: oligonucleotide/oligosaccharide-binding fold containing 2° [*OBFC2A-RARA*, t(2;17)(q32;q21)], general transcription factor II-I [*GTF2I-RARA*, t(7;17)(q11;q21)], fibronectin type III domain-containing protein 3B [*FNDC3B-RARA*, t(3;17)(q26; q21)], nuclear pore complex protein 98 (*NUP98-RARA*, cytogenetic analysis not available), trinucleotide repeat containing 18 (*TNRC18-RARA*, cytogenetic analysis not available), heterogeneous nuclear ribonucleoprotein C (*HNRNPC-RARA*, t(14;17)(q11;q21)), and chromosome X (*X-RARA*, t(X;17)(q28;q12)). In most cases, these AMLs resulted resistant to ATRA and ATO. The only exception is a patient with a FNDC3B-RARA-positive AML, who presented DS after 4 days from ATRA initiation and achieved complete response, but there are no data on the long-term response. Chemotherapy was effective, and CR was achieved in all cases, except for one patient with a *GTF2I-RARA* rearrangement and who experienced early death. Coagulopathy was reported in GTF2I-RARA, FNDC3B-RARA, NUP98-RARA, and TNRC18-RARA-positive AML (70–76).

### RARB Rearrangements

*TBLR1-RARB* rearrangement has been frequently described, with variable cytogenetic features (t(3;3)(q24;q26)/inv.(3)/t(3;10;12)(q26.2;q22;q15)) (77–79). The patients were resistant to ATRA therapy, and no DS occurred, while chemotherapy was effective. One patient presented coagulopathy at the onset of disease. Osumi et al. reported a second type of rearrangement involving RARB, whose partner gene was not detected by whole-genome sequencing [*X-RARB*: t(X;3)(q28;q21)], which occurred in an AML, which was resistant to ATRA and sensitive to chemotherapy (79).

Thus, in patients with RARB rearrangements, chemotherapy seems a valuable option, as it guarantees good results and favorable outcomes, with all patients alive at a mean follow-up of 56.6 months (range, 23–108 months) (**Table 1**).

### RARG Rearrangements

The most commonly described *RARG* rearrangement involves the cleavage and polyadenylation specificity factor subunit 6 (*CPSF6-RARG*, t(12;12)(q13;q15), implicated in solid cancer viability and tumorigenesis and reported in 8 patients (126). Coagulopathy occurred in half of the patients. This variant is not sensitive to treatment with ATRA and ATO, with discrete responses to chemotherapy. Only one case of DS has been described in a patient treated with ATRA+ATO, who died 1 month after diagnosis (27, 77, 80–83).

Five cases of AML with rearrangements between RARG and NUP98 (*NUP98-RARG*, t(11;12)(p15;q13), have been described. *NUP98* is a gene known to fuse with different partner genes in several hematological malignancies (127). Coagulopathy occurred in one patient. ATRA and ATO, alone or in combination, resulted ineffective. One patient presented DS after 14 days from ATRA+ATO initiation, and an *in vitro* study demonstrated ATRA activity (3). Four patients treated with chemotherapy, one of them combined with ATRA, achieved complete response (84–89).

Three other *RARG* partner genes have been reported: *PML* (*PML-RARG* t(12;15)(q13;q22), *NPM1* (*NPM1-RARG-NPM1*, cytogenetic analysis not available), and heterogeneous nuclear ribonucleoproteins C1/C2 (*HNRNPC-RARG*, cytogenetic analysis not available). In these cases, no coagulopathy occurred, and the disease was resistant to ATRA and/or ATO (89–91).

Overall, variant APLs with RARG rearrangements are characterized by poor outcome (more than half of the patients died) and resistance to ATRA and ATO, although these drugs have some effect on blasts, as shown by the reported DSs. Therefore, a combined approach with ATRA ± ATO and chemotherapy may be reasonable.

### Non-RAR Rearrangements

A few cases of APL variants with *RAR*-negative rearrangements have been reported, including myeloid/lymphoid or mixed-lineage leukemia and RNA polymerase II elongation factor *ELL* (*ELL-MLL/MLL-ELL*, t(11;19)(q23;p13.3), *MLL* and *AF1Q* (*MLL-AF1Q*, (t(1;11)(q21;q23), *MLL* and regulation of nuclear pre-MRNA domain containing 2 (*RPRD2-MLL*, t(1;11)(q21;q23), *NPM1* and coiled-coil domain containing 28A (*NPM1-CCDC28A*, cytogenetic analysis not available), *TBC1* domain family member 15, and Ras-associated binding 21 (*TBC1D15-RAB21*, cytogenetic analysis not available) (77, 92).

Most of these genes are known to be involved in leukemogenesis, DNA damage response, and stem cell self-renewal (128–132). All rearrangements have been described in pediatric patients by Zhao et al. *ELL-MLL/MLL-ELL* has been also described in a young woman, the only one who presented with coagulopathy. The patients were successfully treated with a combination therapy (ATRA, ATO, and chemotherapy) (77, 92).

Of note is that a NPM1-CCDC28A fusion transcript has been recently described in an adult patient whose features, however, resembled more an NPM1-mutated AML than APL (93). The similarities between APL and NPM1-mutated AML will be discussed in the dedicated paragraph.

### Complex Rearrangements

Complex rearrangements are translocations involving more than two chromosomes. With the exception of the case reported by Chong et al., all patients presented typical *PML-RARA* fusion transcripts. Compared with the other atypical transcripts, these patients presented features similar to classic APL: a high percentage of coagulopathy (71%), good response to ATRA, ATO, and/or chemotherapy, discrete incidence of differentiation syndrome (29%), and an excellent prognosis (patients alive at a mean follow-up of 23.8 months; range, 3–60 months). This suggests that karyotypic complexity does not affect the characteristics and outcome of ASPL with typical rearrangements (94–100, 133).

Chong et al. described the only complex rearrangement bearing a fusion transcript different from *PML-RARA*. It involves RARA and the tropomyosin receptor kinase-fused gene (*TGF-RARA*, t(3;14;17)(q12;q11;q21), a regulator of intracellular proteins trafficking and fusion partner in solid cancers. This APL variant was also sensitive to ATRA therapy, alone and in combination with chemotherapy, with a favorable outcome. No coagulopathy and DS were observed (94).

### Cases of NPM1-Mutated AML With APL-Like Morphology

Recent studies have shown that, in a discrete proportion of cases (up to 30%), NPM1-mutated AML may present clinical and laboratory features similar to APL.

Mason et al. conducted the study with the largest number of cases and included 42 patients. The immunophenotype commonly found in this subset of AMLs was CD117+MPO+CD34-HLA-DR-CD11b-CD13dim/- (134). Furthermore, in this subset of AMLs, the immunocytochemical pattern of the PML protein may resemble APL (135, 136).

In the case series of Rosainz et al., the morphological features of blasts were similar to APL, with Auer Rods found in 2 of the 5 patients and signs of coagulopathy found in all patients. A possible confounding factor may be the frequent leukocytosis in these patients, a feature uncommon in APL and associated, in AML, with an increased risk of DIC. These patients seem to have a better outcome than AML-NPM1 mutated without APL-like features, with significantly longer relapse-free survival (median 64 *vs*. 9 months) and overall survival (median 81 *vs*. 20 months) in those who achieved CR (137).

Interestingly, Mason et al., examining next-generation sequencing data, found either *TET2* or *IDH1/2* mutation in almost all of these patients (98%) (134). El Hajj et al. and Martelli et al. have recently shown that ATRA and ATO can induce differentiation and apoptosis in NPM-1 blasts, suggesting that this combination may also be effective in NPM1-mutated AMLs (135, 138).

## Final Considerations

Numerous reports in recent years broadened the knowledge on variant APL prognosis and drug resistance profile. With this literature update, we hope to describe these cases in a complete and detailed manner to support the correct classification of the variants, which, although rare, remain a challenge for the clinician.

**Figure 2:**
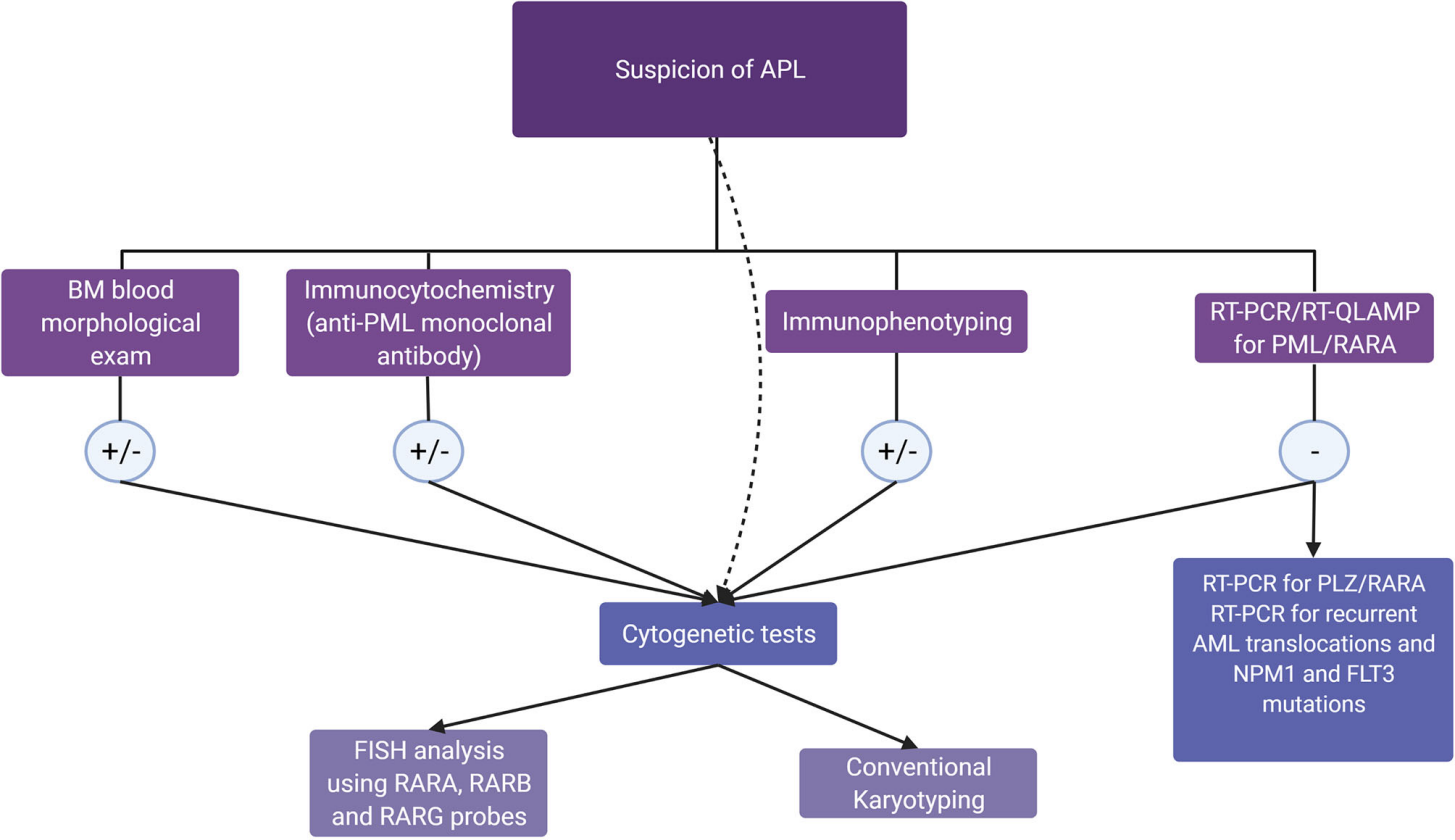
Diagnostic algorithm in the suspicion of acute promyelocytic leukemia (APL) and APL-like acute myeloid leukemias. In patients with morphologic, immunophenotypic, or clinical features raising a suspicion of APL, the guidelines (15) recommend molecular genotyping, which can confirm APL diagnosis in a few hours. If the PML/RARA rearrangement is absent, RT-PCR for recurrent translocations and for PLZF/RARA rearrangement, together with NPM1 and FLT3 mutation testing, should be performed. In all cases, cytogenetic tests, including FISH, allow the diagnostic assessment for RARX rearrangements in 1 to 2 days, while conventional karyotyping will detect karyotype abnormalities in 5–7 days, as recommended by ELN 2017 (139).

A strong suspicion of APL should lead to the prompt initiation of treatment with ATRA and, if the *PML-RARA* transcript is negative, to further diagnostic investigations. **Figure 2** shows a diagnostic algorithm for APL and APL-like AMLs. Frequent monitoring and, possibly, correction of coagulation parameters and blood count are of utmost importance to prevent the onset of coagulopathy. In case of a newly identified transcript, combination therapy may be a valid strategy. Patient management should include careful monitoring of fluid balance, body weight, and any signs of possible SD, which may occur even in the absence of a therapeutic response to ATRA or ATO.

## Author Contributions

LG, TO, and MTV composed, edited, and finalized the review. ST designed the figure. EF, MD, AR, GF, PP, and MCR reviewed the text. All authors contributed to the article and approved the submitted version.

## Funding

Grant from Ministero della Salute, Rome, Italy (Finalizzata 2018, NET-2018-12365935, Personalized medicine program on myeloid neoplasms: characterization of the patient's genome for clinician decision making and systematic collection of real world data to improve quality and health care) to MTV.

## Conflict of Interest

The authors declare that the review was conducted in the absence of any commercial or financial relationships that could be construed as a potential conflict of interest.

## Publisher’s Note

All claims expressed in this article are solely those of the authors and do not necessarily represent those of their affiliated organizations, or those of the publisher, the editors and the reviewers. Any product that may be evaluated in this article, or claim that may be made by its manufacturer, is not guaranteed or endorsed by the publisher.
